# Modeling biological age using blood biomarkers and physical measurements in Chinese adults

**DOI:** 10.1016/j.ebiom.2023.104458

**Published:** 2023-02-07

**Authors:** Lu Chen, Yiqian Zhang, Canqing Yu, Yu Guo, Dianjianyi Sun, Yuanjie Pang, Pei Pei, Ling Yang, Iona Y. Millwood, Robin G. Walters, Yiping Chen, Huaidong Du, Yongmei Liu, Sushila Burgess, Rebecca Stevens, Junshi Chen, Zhengming Chen, Liming Li, Jun Lv, China Kadoorie Biobank Collaborative Group

**Affiliations:** a Department of Epidemiology & Biostatistics, School of Public Health, Peking University, Beijing, 100191, China; b Peking University Center for Public Health and Epidemic Preparedness & Response, Beijing, 100191, China; c Fuwai Hospital Chinese Academy of Medical Sciences, Beijing, China; d Medical Research Council Population Health Research Unit at the University of Oxford, Oxford, United Kingdom; e Clinical Trial Service Unit & Epidemiological Studies Unit (CTSU), Nuffield Department of Population Health, University of Oxford, United Kingdom; f Qingdao Centers for Disease Control and Prevention (CDC), Qingdao, China; g China National Center for Food Safety Risk Assessment, Beijing, China

**Keywords:** Biological age, Blood biomarkers, Physical measurements, Mortality, Cardiovascular health

## Abstract

**Background:**

This study aimed to: 1) assess the associations of biological age acceleration based on Klemera and Doubal's method (KDM-AA) with long-term risk of all-cause mortality; and 2) compare the association of KDM-AA with all-cause mortality among participants potentially at different stages of the cardiovascular disease (CVD) continuum.

**Methods:**

The present study was based on a subpopulation of the China Kadoorie Biobank, with baseline survey during 2004–08. A total of 12,377 participants free of ischemic heart disease, stroke, or cancer at baseline were included, in which 8180 participants were identified to develop major coronary event (MCE), ischemic stroke (IS), intracerebral hemorrhage (ICH) or subarachnoid hemorrhage (SAH), and 4197 remained free of these cardiovascular diseases before 1 January 2014. These participants were followed up until 1 Jan 2018. KDM-AA was calculated by regressing biological age measurement, which was constructed based on baseline 16 physical and 9 biochemical markers using Klemera and Doubal's method, on chronological age. We estimated the associations of KDM-AA with the mortality risk using the hazard ratio (HR) and 95% confidence interval (CI) from Cox proportional hazard models. We assessed discrimination performance by Harrell's C-index and net reclassification index (NRI).

**Findings:**

The participants who developed MCE (mean KDM-AA = 0.1 year, standard deviation [SD] = 1.6 years) or ICH/SAH (0.3 ± 1.5 years) during subsequent follow-up showed accelerated aging at baseline compared to those of IS (0.0 ± 1.2 years) and control (−0.3 ± 1.3 years) groups. The KDM-AA was positively associated with long-term risk of all-cause mortality (HR = 1.20; 95% CI: 1.17, 1.23), and the association was robust for participants potentially at different stages of the CVD continuum. Adding KDM-AA improved mortality prediction compared to the model only with sociodemographic and lifestyle factors in whole participants, with the Harrell's C-index increasing from 0.813 (0.807, 0.819) to 0.821 (0.815, 0.826) (NRI = 0.011; 95% CI: 0.003, 0.019).

**Interpretation:**

In this middle-aged and elderly Chinese population, the KDM-AA is a promising measurement for biological age, and can capture the difference in cardiovascular health and predict the risk of all-cause mortality over a decade.

**Funding:**

This work was supported by 10.13039/501100001809National Natural Science Foundation of China (82192904, 82192901, 82192900, 81941018). The CKB baseline survey and the first re-survey were supported by a grant from the 10.13039/501100017647Kadoorie Charitable Foundation Hong Kong. The long-term follow-up is supported by grants from the UK Wellcome Trust (212946/Z/18/Z, 202922/Z/16/Z, 104085/Z/14/Z, 088158/Z/09/Z), grants (2016YFC0900500) from the National Key R&D Program of China, National Natural Science Foundation of China (81390540, 91846303), and Chinese Ministry of Science and Technology (2011BAI09B01).

**Research in context**

**Evidence before this study**

The aging pace in a population of the same chronological age varies between individuals. Biological age (BA), constructed by physical and biochemical markers, may help define whether aging is accelerated or delayed relative to chronological age. Also, this type of BA measurement tends to capture physiological alterations earlier than specific phenotypes and can be obtained at a lower cost than omics-based indicators. Klemera and Doubal's method (KDM), as one of the BA estimation algorithms, has shown superiority in predicting BA and mortality. Most studies on KDM-BA were conducted in western populations, and only a few in Asian populations recently.

**Added value of this study**

The present study was based on a subpopulation of the China Kadoorie Biobank cohort (CKB). A total of 12,377 participants free of ischemic heart disease, stroke, or cancer at baseline were included, in which some participants developed various subtypes of cardiovascular diseases (CVD) thereafter, and the others remained free of these CVDs for up to 10 years of follow-up. This study constructed BA based on baseline 16 physical and 9 biochemical markers using KDM and further calculated the biological age acceleration (KDM-AA).

The present study first suggests that the participants who subsequently developed major coronary event, ischemic stroke, or hemorrhagic stroke during follow-up showed varying degrees of accelerated aging at baseline. We exploited the advantages of CKB in outcome accuracy and examined the associations of the KDM-AA with long-term risk of all-cause mortality. Finally, we offered evidence that the association of KDM-AA with all-cause mortality was robust for participants potentially at different stages of the CVD continuum and those with different sociodemographic characteristics and lifestyles. The KDM-AA could improve the discrimination performance of prediction models.

**Implications of all the available evidence**

This study provides the comprehensive association analysis of KDM-AA with all-cause mortality by subgroups and also first uses general BA measurement for capturing the difference in cardiovascular health. The present study suggests that the KDM-AA may be a useful indicator for risk stratification, especially in a relatively healthy population.

## Introduction

Aging is the ‘common soil' of age-related chronic diseases, the decline in activities of daily living, and death. Chronological age (CA) is a convenient measurement of the aging state of individuals. However, the aging pace in a population of the same CA varies between individuals, and variability in cognitive function^1^ and health status^2^ also increases with CA. Therefore, biological age (BA) was proposed to define whether aging was accelerated or delayed relative to chronological age.^3^

A variety of BA measurements have emerged recently, such as omics-based indicators (e.g., telomere length,^4^ Horvath's clock,^5^ and Hannum's clock^6^), clinical indicators (e.g., phenotypic age^7^ and BA measurement constructed under Klemera and Doubal's method [hereafter KDM-BA]),^8^ and phenotypic indicators (e.g., frailty phenotype,^9^ frailty index,^10^ and functional aging index^11^). There is yet no gold standard for measuring BA. By contrast, clinical indicators, usually constructed by physical and biochemical markers, tend to capture physiological alterations earlier than specific phenotypes and can be obtained at a lower cost than omics-based indicators.

Klemera and Doubal's method (KDM), as one of the BA estimation algorithms, has shown superiority in predicting BA and mortality.^12^^,^^13^ Although the number and types of markers included in KDM-BA construction differed slightly across studies, KDM-BA showed consistent associations with aging outcomes.^8^^,^^12^^,^^14^ Most studies on KDM-BA were conducted in western populations, and only a few in Asian populations recently.^15^, ^16^, ^17^, ^18^ A study in the Chinese population indicated that KDM-BA was predictive of all-cause mortality in two cohorts.^15^

The present study was based on a subpopulation of the China Kadoorie Biobank (CKB), a cohort of middle-aged and older adults with a mean follow-up period of approximately 10 years. We aimed to assess the associations of biological age acceleration (AA) based on KDM-BA (KDM-AA) at baseline with long-term risk of all-cause mortality. Biological AA was first seen in studies on DNA methylation age^19^ and is a deviation of BA from CA, reflecting the additional value of BA over CA. This study included over 10,000 participants free of ischemic heart disease (IHD), stroke, or cancer at baseline. Some participants had different subtypes of cardiovascular disease (CVD) diagnosed shortly after baseline and were more likely to have been in the stage of subclinical CVD at baseline, while others remained CVD-free after longer follow-up. This gives us an opportunity to compare the association of KDM-AA with all-cause mortality among participants potentially at different stages of the CVD continuum.

## Methods

### Study population

The baseline survey of the CKB cohort was launched in five urban and five rural areas during 2004–2008, enrolling a total of more than 0.5 million participants aged 30–79. The baseline design and the profile of CKB participants were described in detail previously.^20^^,^^21^ All participants provided written informed consent. The study protocol was approved by the Ethical Review Committee of the Chinese Center for Disease Control and Prevention (CDC, Beijing, China) and the Oxford Tropical Research Ethics Committee at the University of Oxford (Oxford, UK).

A subpopulation of CKB cohort was selected to measure a series of clinical biochemistry biomarkers. All participants who reported having doctor-diagnosed IHD, stroke, or cancer or self-reported statin users at baseline were first excluded from the selection process. The following participants with incident events documented during follow-up till the date of 1 January 2014 were identified: (1) major coronary event (MCE): fatal IHD (the 10th revision of the International Classification of Diseases, ICD-10: I20–I25) or nonfatal myocardial infarction (MI, I21–I23); (2) ischemic stroke (IS, I63 or I69.3); (3) intracerebral hemorrhage (ICH, I61 or I69.1) or subarachnoid hemorrhage (SAH, I60 or I69.0). Another group of participants was selected from those free of cardiovascular events till 1 January 2014, called the control group. Finally, 18,172 participants (the control group: n = 6341; MCE group: 1319; IS group: 5467; ICH/SAH group: 5045) were included for biochemistry tests.

The vital and disease status and hospitalization were updated for all participants as the follow-up continued. Due to updated outcome definition and case adjudication by expert physicians, the status of a few cases and controls aforementioned might be changed.^22^ However, the original grouping was retained in the present analysis.

### Baseline data collection

At the baseline survey, trained staff interviewed participants to collect sociodemographic characteristics (e.g., age, sex, educational attainment), lifestyle factors, and personal medical and family histories. Information on tobacco smoking included smoking status (never, ever, or currently) and the main reason for smoking cessation in quitters. Questions about alcohol drinking included drinking frequency in the past 12 months (never, occasionally, monthly, weekly, or daily), the quantity of alcohol consumed in weekly drinkers, and past drinking status in non-weekly drinkers. We inquired about the frequency of dietary intake in the past 12 months (never/rarely, monthly, 1–3 days/week, 4–6 days/week, or daily) via a validated qualitative food frequency questionnaire, which covered 12 major food groups.^23^ The level of physical activity was assessed by multiplying the metabolic equivalent tasks (METs) values of activities by the typical duration of corresponding activities in hours per day and summing them together based on the 2011 update of compendium of physical activities.^24^

In the present study, we defined risky lifestyle factors (tobacco smoking, alcohol drinking, dietary habits, and physical activity) according to previous findings^25^, ^26^, ^27^ and adapted them to the present study. The risky dietary habits were defined according to the following criteria: eating vegetables less than daily, eating fruits less than daily, eating red meat daily or less than weekly, eating soybean <4 days per week, and eating fish less than weekly.^25^, ^26^, ^27^ One point was for one food item, and the total score was the risky dietary score, ranging from 0 to 5. The risky lifestyle factors included: currently smoking (including quitting due to illness); daily intake of pure alcohol ≥30 g for males, ≥15 g for females, or past drinking; the risky dietary score of 4–5 points; and the total physical activity level ranking in the lower age- (<50 years, 50–59 years, and ≥60 years) and sex-specific half. The risky lifestyle score was the number of the risky lifestyle factors, ranging from 0 (healthiest) to 4 (riskiest). Participants who scored 3 or 4 were categorized into the risky group.

Physical measurements included height, weight, waist circumference, hip circumference, heart rate, systolic blood pressure (SBP), diastolic blood pressure (DBP), and forced expiratory volume in 1 s (FEV1). Standing height and body weight was measured by a height meter and a TANITA TBF-300GS body fat meter, respectively. Waist circumference and hip circumference measurements were taken using a soft tape. Blood pressure and heart rate measurement were taken twice for each participant by a UA-779 digital sphygmomanometer. A third measurement was performed if the difference between the first two SBP measurements were greater than 10 mmHg, and the latter two readings were recorded. FEV1 was assessed by a spirometer, and two successful blows were recorded. We utilized mean values of heart rate, SBP, and DBP and the larger FEV1 in our construction of KDM-BA. All measurements were carried out under standard operating instructions. Previous literature can be referred to for details.^28^

### Clinical biomarker measurement

For each participant, a 10 mL blood sample was collected at baseline without fasting requirement but with the time since last meal recorded. Random plasma glucose (RPG) was tested on-site using SureStep Plus meters (Lifescan, Johnson & Johnson). The participants with RPG of 7.8–11.0 mmol/L were invited to retest their fasting plasma glucose (FPG) the next morning. Clinical biochemistry tests were performed for 1 mL baseline plasma samples at the Wolfson Laboratory (Clinical Trial Service Unit & Epidemiological Studies Unit, UK). All biomarkers listed in Supplementary Table S1 were assayed using AU680 clinical chemical analyzer (Beckman Coulter Incorporation, UK), except that cystatin C was measured by BN Prospec nephelometer analyzer (Siemens, UK).^29^ All tests were performed using the manufacturer's standard reagents, calibrators, and settings.

A subset of participants was selected for high-throughput targeted NMR spectroscopy measurements at the Brainshake Laboratory (Kuopio, Finland).^30^ There was an overlapping of nine traits (albumin, creatinine, total cholesterol, low density lipoprotein cholesterol, high density lipoprotein cholesterol, total triglyceride, apolipoprotein A1, apolipoprotein B, and RPG) between the clinical biochemistry tests and the NMR spectroscopy, with good agreement between measurements (r = 0.77–0.98).

### Construction of biological age and age acceleration

A list of markers, including blood biomarkers and physical measurements, was considered in the construction of KDM-BA (Supplementary Table S2). For participants who reported taking antihypertensive medication (n = 2160), we added 15 and 10 mmHg to measured SBP and DBP, respectively.^31^ For participants with both random and fasting blood glucose levels measured, the fasting blood glucose value was used for the analysis (n = 657). According to distributions of markers, the log-transformation was used for biomarkers except for plasma albumin and all physical measurements.

According to the procedure proposed by Klemera,^8^ Levine,^12^ and a previous study,^32^^,^^33^ we constructed KDM-BA by sex. First, we calculated the Pearson's correlation coefficient of each marker with CA, and those with coefficients above a certain threshold (>0.10) were included in the construction of KDM-BA. The markers which showed a non-monotonic relationship with CA were excluded. Finally, sixteen markers remained in the calculation for both sexes. The remaining markers were then transformed into principal components (PCs) using principal component analysis based on the correlation matrix, which were further used to calculate the KDM-BA according to the Klemera and Doubal's method.^8^^,^^12^^,^^32^^,^^33^ KDM-AA was calculated by regressing KDM-BA on CA in the present study. The formula of calculation is in Supplementary methods.

### Outcome ascertainment

During long-term follow-up, the vital status was ascertained through local Disease Surveillance Point (DSP) death registries using unique personal identification numbers, supplemented by annual checks with local residential records and active confirmation by contacting local communities or relatives.^21^ The causes of death, mainly derived from official death certificates, were supplemented by reviewing medical records and verbal autopsy based on symptoms and signs provided by informants (usually family members).^34^ The disease incidence was obtained by establishing linkage to the local disease registry system and national inpatient health insurance claim data in which >97% of participants were covered (these procedures also yielded a few additional deaths that had not been identified through death registries).^34^ All participants were followed until death or loss to follow-up. The data for the present analysis was censored on 1 Jan 2018.

### Statistical analysis

Supplementary Fig. S1 illustrates the study design. Among 18,172 participants who had biochemical tests, we excluded (1) those with blood samples lipemic, icteric, haemolysed, or turbid (n = 3726); (2) those with values marked as under limit of detection (n = 1452); (3) those with missing values on any of the markers listed in Supplementary Table S2 (n = 167); (4) outliers, namely the participants with any marker lying outside of four standard deviations (SD, n = 450), leaving a total of 12,377 participants in the analysis.

We conducted the following analyses in whole participants and by four groups (control, MCE, IS, and ICH/SAH) separately, where appropriate. Baseline characteristics were compared among four groups using linear regression models for continuous variables and logistic regression models for categorical variables. The contrast was also made between included versus excluded participants. To assess the accuracy of KDM-BA in predicting CA of the aging model, we calculated the Pearson's correlation coefficient between KDM-BA and CA and root mean square error (RMSE) by regressing CA on KDM-BA.

We examined the association of KDM-AA with the risk of all-cause mortality using the hazard ratio (HR) and 95% confidence interval (CI) from Cox proportional hazard models. Cox models were stratified jointly by age groups in the 5-year interval, 10 study areas, and four groups (control, MCE, IS, and ICH or SAH), where appropriate, with CA as the underlying time scale. Models were adjusted for sex, fasting status (<8 or ≥8 h), and educational attainment (primary school and below, middle or high school, college or university). No major violations of the proportional hazards assumption for KDM-AA were identified. Our study further explored associations of KDM-AA with all-cause mortality stratified by sociodemographic (age groups, sex, urban or rural areas) and lifestyle factors (smoking, alcohol drinking, dietary habits, physical activity level, combined lifestyle factors) at baseline. The interactions between KDM-AA and the stratifying variables were tested using likelihood ratio tests by comparing models with and without product terms.

We also evaluated Cox models including CA only or KDM-AA additionally for predicting 10-year mortality risk by Harrell's C-index and net reclassification index (NRI) for the traditional 50% threshold.^35^^,^^36^ The basic models used time-on-study as the time scale and were stratified jointly by 10 study areas and four groups (control, MCE, IS, and ICH or SAH), adjusting for the same covariates as above, as well as urban or rural areas and lifestyle factors (smoking, alcohol drinking, dietary habits, physical activity level).

We performed sensitivity analysis by restricting the construction of KDM-BA by sex in the control group, as mentioned above. We then calculated PCs and KDM-BA for the case groups by sex using loadings of markers and weights of CA and PCs, which were derived from the control analysis. Considering the differences in standard deviation (SD) characteristics of KDM-AA between the primary and sensitivity analysis, we standardized KDM-AA by dividing SD to make the association estimates comparable. We also performed sensitivity analysis by excluding participants who reported taking antihypertensive medication.

We used *TrueTrait* function from WGCNA package in R (version 4.0.3) for the construction of KDM-BA and Stata (version 15.0) for other statistical analyses. Statistical significance was defined by the two-sided *P*-values <0.05.

This work was supported by 10.13039/501100001809National Natural Science Foundation of China (82192904, 82192901, 82192900, 81941018). The CKB baseline survey and the first re-survey were supported by a grant from the 10.13039/501100017647Kadoorie Charitable Foundation in Hong Kong. The long-term follow-up is supported by grants from the UK 10.13039/100010269Wellcome Trust (212946/Z/18/Z, 202922/Z/16/Z, 104085/Z/14/Z, 088158/Z/09/Z), grants (2016YFC0900500) from the National Key R&D Program of China, 10.13039/501100001809National Natural Science Foundation of China (81390540, 91846303), and 10.13039/100007225Chinese Ministry of Science and Technology (2011BAI09B01).

### Role of funding source

The funders had no role in the study design, data collection, data analysis and interpretation, writing of the report, or the decision to submit the article for publication.

## Results

The four groups based on the disease status till the date of 1 January 2014 differed in their sociodemographic characteristic, lifestyle habits, and personal and family medical histories (Table 1). For example, participants in MCE and IS groups were younger than those in the control and ICH/SAH groups. IS and ICH/SAH groups had higher proportions of excessive alcohol drinking among men. All three case groups had a higher prevalence of hypertension and diabetes. Baseline characteristics between included versus excluded participants were similar in quantity except for the prevalence of diabetes (Supplementary Table S3). The mean values (SD) of blood biomarkers and physical measurements across the groups are shown in Supplementary Table S4.

**Table 1:** Baseline characteristics of participants according to four separate groups (N = 12,377).

	All	Control	MCE	IS	ICH or SAH	*P*-value
Number of participants	12,377	4197	876	3837	3467	
Sociodemographic characteristic						
Age, year	57.0	58.4	54.2	53.7	59.6	<0.001
Female, %	6218 (50.2)	2013 (48.0)	377 (43.0)	2137 (55.7)	1691 (48.8)	<0.001
Urban, %	3690 (29.8)	974 (23.2)	277 (31.6)	1681 (43.8)	758 (21.9)	<0.001
Middle school or above, %	38.3	39.1	35.7	38.6	37.6	0.051
Lifestyle						
Current smoking^a^, %						
Male	69.6	67.1	77.8	72.1	67.7	<0.001
Female	4.0	3.6	6.8	4.7	3.8	0.048
Excessive alcohol drinking^b^, %						
Male	23.7	19.9	22.1	27.2	25.7	<0.001
Female	1.8	1.6	0.8	1.7	2.5	0.078
Dietary habits, %						
Eating fresh vegetables: not daily	7.1	6.7	7.0	7.6	7.2	0.492
Eating fresh fruits: not daily	87.1	86.1	89.8	86.5	88.6	<0.001
Eating red meat: daily or less than weekly	49.7	48.1	51.4	50.4	50.2	0.069
Eating fish: less than weekly	67.0	66.5	71.1	65.8	68.3	<0.001
Eating soybean: <4 days/week	90.8	90.7	91.5	90.3	91.4	0.420
Total physical activity, MET-h/d	18.8	19.3	18.9	18.7	18.2	0.002
Fasting time, h	4.7	4.6	4.7	4.8	4.7	0.181
Prevalent hypertension, %	55.0	40.0	57.3	58.2	69.3	<0.001
Prevalent diabetes, %	6.7	3.7	11.1	9.3	6.2	<0.001
Medication of, %						
Antihypertensives	17.5	10.5	19.2	20.1	22.5	<0.001
Antidiabetics	2.4	1.5	4.6	3.3	1.8	<0.001
Family history of, %						
Heart disease	3.5	3.1	5.3	3.6	3.1	0.006
Stroke	21.6	19.2	21.5	22.7	23.2	<0.001
Cancer	16.3	17.9	16.7	16.6	13.8	<0.001
Self-rated good health, %	41.4	45.6	38.6	38.8	40.0	<0.001

MET, metabolic equivalent of task; MCE: major coronary event; IS: ischemic stroke; ICH: intracranial hemorrhage; SAH: subarachnoid hemorrhage.

Sex and urban regions are presented as n (%). Other values are means or percentages with adjustment for age, sex, and study regions, where appropriate.

a Former smoker who had stopped smoking for illness was categorized into the current smoker.

b Daily intake of pure alcohol: ≥30 g for males, ≥15 g for females; former drinker was categorized into excessive alcohol drinker.

**Fig. 1:**
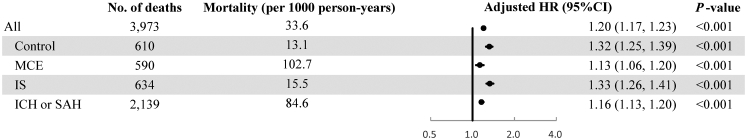
**Associations of per 1-year increment of KDM age acceleration with all-cause mortality in all participants and four separate groups.** KDM: Klemera and Doubal's method; MCE: major coronary event; IS: ischemic stroke; ICH: intracranial hemorrhage; SAH: subarachnoid hemorrhage; HR: hazard ratio. Cox models adjusted for sex, fasting status, and educational attainment, stratified jointly by age groups in the 5-year interval, study areas, and four groups.

MCE group had the highest mortality rate (102.7 per 1000 person-years), followed by ICH/SAH group (84.6 per 1000 person-years), IS group (15.5 per 1000 person-years), and the control group (13.1 per 1000 person-years) (Fig. 1). As shown in Table 2, the median (interquartile range, IQR) time to the first diagnosis of the corresponding diseases was 4.4 (3.9) years for the MCE group, 4.6 (3.8) years for IS group, and 4.1 (3.6) years for ICH/SAH group. The median (IQR) time to death for the above three case groups is 5.8 (7.4) years, 11.0 (2.1) years, and 7.2 (7.1) years, respectively.

**Table 2:** Summary of CVD events during follow-up according to four separate groups.

	Control	MCE	IS	ICH or SAH
Number of events				
CVD cases	451	807	3562	3342
MCE cases	86	803	143	97
IS cases	321	25	3547	601
ICH or SAH cases	74	2	213	3316
Incidence, per 1000 person-years				
CVD cases	9.8	201.7	194.8	227.3
MCE cases	1.9	200.2	3.5	3.8
IS cases	7.0	4.4	193.5	25.9
ICH or SAH cases	1.6	0.3	5.3	224.0
Median (IQR) time to events, year				
Deaths/loss to follow-up/1 Jan 2018	11.6 (1.9)	5.8 (7.4)	11.0 (2.1)	7.2 (7.1)
CVD cases	11.5 (2.1)	4.4 (3.9)	4.6 (3.8)	4.1 (3.6)
MCE	11.6 (1.9)	4.4 (3.9)	11.0 (2.1)	7.2 (7.1)
IS cases	11.5 (2.0)	5.8 (7.3)	4.6 (3.8)	6.2 (6.7)
ICH or SAH cases	11.6 (1.9)	5.8 (7.4)	11.0 (2.1)	4.1 (3.6)

IQR: interquartile range; CVD: cardiovascular disease; MCE: major coronary event; IS: ischemic stroke; ICH: intracranial hemorrhage; SAH: subarachnoid hemorrhage.

CVD cases correspond to any first occurrence of MCE, IS (ICD10: I63, I69.3), ICH (I61, I69.1), or SAH (I60, I69.0) till the censoring date of 1 Jan 2018. MCE refers to nonfatal myocardial infarction (I21-23) or fatal IHD (I20-25) here.

The distribution of KDM-AA showed statistically significant differences across groups (*P* < 0.001) (Table 3). The control group exhibited decelerated aging as a whole (KDM-AA [SD] = -0.3 [1.3] year), while the MCE (0.1 [1.6] year) and ICH/SAH (0.3 [1.5] year) groups displayed accelerated aging. The correlations between KDM-BA and CA were extremely high in the whole population and across groups (r = 0.98–0.99). The error of KDM-BA in predicting CA lies in the range from 1.24 years to 1.52 years. The control-derived KDM-AA demonstrated similar distribution but smaller difference and SD among groups (Supplementary Table S5).

**Table 3:** Summary of CA and KDM-BA-related measurements for participants according to four separate groups.

	All	Control	MCE	IS	ICH or SAH	*P*-value
CA, year	57.0 (10.5)	58.4 (11.0)	54.2 (8.6)	53.7 (9.4)	59.6 (10.4)	<0.001
KDM-BA, year	57.0 (10.6)	58.2 (11.1)	54.3 (8.8)	53.6 (9.5)	59.9 (10.5)	<0.001
KDM-AA, year	0.0 (1.4)	−0.3 (1.3)	0.1 (1.6)	0.0 (1.2)	0.3 (1.5)	<0.001
RMSE, year	1.39	1.33	1.52	1.24	1.50	–
Pearson's correlation coefficient between KDM-BA and CA	0.99	0.99	0.98	0.99	0.99	–

KDM: Klemera and Doubal's method; CA: chronological age; BA: biological age; AA: age acceleration; RMSE: root mean square error; MCE: major coronary event; IS: ischemic stroke; ICH: intracranial hemorrhage; SAH: subarachnoid hemorrhage.

CA, KDM-BA, and KDM-AA measurements are presented as means (SD).

In the control group, accelerated aging was associated with an increased risk of mortality, with the adjusted HR (95% CI) for per 1-year increment of KDM-AA equal to 1.32 (1.25, 1.39) (Fig. 1). The HRs (95% CIs) for MCE, IS, and ICH/SAH groups were 1.13 (1.06, 1.20), 1.33 (1.26, 1.41), and 1.16 (1.13, 1.20), respectively. When the four groups merged into one, the positive association of KDM-AA with mortality remained robust (HR = 1.20; 95% CI: 1.17, 1.23). The associations of KDM-AA with mortality were also observed in all stratification analyses by sociodemographic and lifestyle factors at baseline. The strength of the association of KDM-AA with the risk of mortality was stronger in the participants aged <60, female, non-smokers, or non-excessive alcohol drinkers than in their counterparts (Supplementary Table S6).

Compared with models with CA included, the Harrell's C-indexes of models with KDM-AA included increased across all groups (increments = 0.008–0.020), and point estimates of NRIs varied from 0.011 to 0.073. The Harrell's C-indexes for models including only CA and both CA and KDM-AA in whole participants were 0.813 (95% CI: 0.807, 0.819) and 0.821 (0.815, 0.826), respectively (Table 4).

**Table 4:** Harrell's C-index and NRI for CA and KDM-AA models in all participants and four separate groups.

	Harrell's C-index (95% CI)		NRI (95% CI)
	Basic model + CA	Basic model + CA + KDM-AA	
All	0.813 (0.807, 0.819)	0.821 (0.815, 0.826)	0.011 (0.003, 0.019)
Control	0.775 (0.758, 0.793)	0.794 (0.777, 0.811)	0.073 (0.048, 0.099)
MCE	0.641 (0.619, 0.664)	0.652 (0.629, 0.674)	0.019 (−0.020, 0.058)
IS	0.745 (0.726, 0.765)	0.765 (0.747, 0.784)	0.036 (0.008, 0.065)
ICH or SAH	0.665 (0.653, 0.677)	0.676 (0.664, 0.687)	0.021 (0.002, 0.040)

NRI: net reclassification index; CA: chronological age; KDM: Klemera and Doubal's method; CI: confidence interval; AA: age acceleration; MCE: major coronary event; IS: ischemic stroke; ICH: intracranial hemorrhage; SAH: subarachnoid hemorrhage.

Basic Cox models were adjusted for sex, fasting status, educational attainment, urban or rural areas, and lifestyle factors (smoking, alcohol drinking, dietary habits, physical activity level), stratified jointly by study areas and four groups.

In the sensitivity analysis of restricting the construction of KDM-BA in the control group, the association estimates between KDM-AA and all-cause mortality in all participants and four separate groups were comparable to the HRs in the primary analysis and showed similar relative differences between groups (Supplementary Table S7). When the analysis was restricted to participants without taking antihypertensive medication, the results were consistent with the primary analysis, with most association estimates even slightly higher (Supplementary Table S8).

## Discussion

This study constructed BA using KDM in a Chinese adult population, showing that the KDM-BA fit CA well. The participants who developed MCE or ICH/SAH during subsequent follow-up showed accelerated aging at baseline. The KDM-AA was positively associated with long-term risk of all-cause mortality and could improve the discrimination performance of models. The association of KDM-AA with all-cause mortality was robust for participants potentially at different stages of the CVD continuum and those with different sociodemographic characteristics and lifestyles.

In the present study, the KDM-BA was highly correlated with CA, with a small prediction error, partly because CA was included as one of the composite markers in the calculation. However, the associations of KDM-AA with death risk remained in our population of a wide age range after ruling out the influence of CA. Also, the discrimination performance with the additional inclusion of KDM-AA in models was better than that including CA. In previous studies of western populations, the association estimates of per 1-year increment of KDM-AA with all-cause mortality were around 1.10.^12^^,^^32^^,^^37^^,^^38^ A prospective study of 8119 Chinese participants aged 20–79 years between 2009 and 2015 waves utilized 12 biomarkers covering immune, cardiometabolic, liver, and renal function to calculate KDM-BA, demonstrating that per 1-year increment of KDM-AA was associated with a 14% (HR = 1.14; 95% CI: 1.08, 1.19) increased risk of all-cause mortality.^15^ A longitudinal study of 951 Taiwanese aged ≥54 years during 2000–2006 found KDM-BA improved discrimination of mortality over a basic model including CA and sex (base-model Harrell's C-index = 0.725; KDM-BA-model Harrell's C-index = 0.755).^16^

Previous studies have shown discrepancies in KDM-BA among participants at different stages of the disease course. A U.S. population study showed a difference in the KDM-BA among participants with diabetes, prediabetes, and age- and sex-matched controls.^39^ The study found that participants without diabetes were 2.69 years younger than those with prediabetes and 5.73 years younger than those with diabetes in KDM-BA.^39^ A study based on two Chinese adult cohorts found that participants with more chronic diseases over six years had higher baseline KDM-AA.^15^ In the present study, the constructed KDM-AA included biochemical markers covering liver and renal function, lipids, inflammation, and metabolism, and physical measurements related to cardiovascular, respiratory, and metabolic status. We found that even in the absence of IHD, stroke, or cancer at baseline, KDM-AA also somewhat distinguished individuals who might have been in the subclinical stage of CVD. Participants who subsequently developed the ICH/SAH or MCE showed an acceleration of aging, and they also had the earliest median time to subsequent onset of diseases compared to other groups. These findings support that KDM-AA as a composite measurement can capture multisystem aging, including vascular senescence.^40^ However, the association estimates between KDM-AA and death risk were slightly weaker in the ICH/SAH and MCE groups than in control and IS groups. It might be attributed to the already high death risk in these two groups, characterized by high case fatality and the earlier time to death, leaving relatively low additional predictive value for KDM-AA.

In our study, the association strength of KDM-AA with death risk was stronger in relatively healthy individuals, namely young adults, females, non-smokers, or non-excessive alcohol drinkers. These results were consistent with the above finding of stronger association in the control group, possibly with a similar explanation. A study based on the Dunedin Study birth cohort of young adults followed up over 12 years, from ages 26 to 38, showed variation in the pace of aging.^41^ Other studies, either constructing phenotypic age^42^ or KDM-AA,^15^ consistently found that the BA measurements could stratify the risk of death in participants with normal BMI and no defined diseases, with the association stronger in the young age group.^42^

This study has some advantages. The large sample size and long follow-up ensured robust results and an opportunity to observe the long-term effect of KDM-AA. The study population was characterized by a wide age range and various sociodemographic and lifestyle characteristics, making the conclusion more generalizable. Also, we could further analyze whether the performance of KDM-AA was consistent in the populations with diverse characteristics. The study included participants potentially at different stages of the CVD continuum, providing an opportunity to explore the differences in the performance of KDM-AA in different subpopulations.

Some limitations should be considered. First, the study participants were from a subpopulation of large prospective cohort study, and there existed an overrepresentation of people with poor cardiovascular health. However, all measurements were taken at baseline when all included participants were free of CVDs or cancer. All CVD cases were newly diagnosed during the follow-up. We restricted the construction of KDM-BA in the control group and then derived KDM-BA for the case groups, and there was no significant change in the results. Second, the composition of KDM-BA was limited to the available clinical markers and may miss some markers more relevant to aging, such as blood cell count. Although the types and number of clinical markers included in this study differed from previous studies, the associations of KDM-AA with mortality were consistent. This also suggests that the construction of KDM-BA doesn't need the same clinical markers as long as the markers cover a wide range of biological systems.

In conclusion, the KDM-AA, based on physical and biochemical markers, was a promising BA measurement in the middle-aged and elderly Chinese. The KDM-AA captured the difference in cardiovascular health and predicted the risk of all-cause mortality over a decade. It may be a useful indicator for risk stratification, especially in a relatively healthy population. The idea of constructing KDM-AA could be learned to maximize the use of data platforms for routine clinical practice to achieve effective and efficient risk stratification and early public health intervention.

## Contributors

JL conceptualised and designed the paper. LL, ZC, and JC, as members of the CKB steering committee, designed and supervised the conduct of the whole study, obtained funding, and together with CY, YG, PP, LY, IM, RW, YC, HD, YL, SB, and RS acquired the data. LC, YZ, CY, YP, JL accessed and verified the data. LC and YZ analyzed the data and LC drafted the manuscript. JL contributed to the interpretation of the results and critical revision of the manuscript for important intellectual content. All authors contributed to and approved the final manuscript, and were responsible for the decision to submit the manuscript. JL is the study guarantor.

## Data sharing statement

The access policy and procedures for the dataset of this study are available at www.ckbiobank.org.

## Declaration of interests

The authors declare that there is no conflict of interest.
