# Supplementary material for: Modeling biological age using blood biomarkers and physical measurements in Chinese adults
Source: eBioMedicine. 2023 Feb 7;89:104458. doi: 10.1016/j.ebiom.2023.104458 (PMC9941058; doi:10.1016/j.ebiom.2023.104458)
Supplement: Supplementary material [file mmc1.docx]

Supplementary File

Supplementary material for the manuscript entitled “Modeling biological age using blood biomarkers and physical measurements in Chinese adults”

**Catalogue**

[Members of the China Kadoorie Biobank collaborative group 2](#_Toc119915084)

[Supplementary methods 3](#_Toc119915085)

[Supplementary figure 1. Study overview 4](#_Toc119915086)

[Supplementary table 1. Summary of reagents, calibrators, and settings in clinical biochemistry tests 5](#_Toc119915087)

[Supplementary table 2. Pearson’s correlation coefficients between CA and blood biomarkers or physical measurements stratified by sex 7](#_Toc119915088)

[Supplementary table 3. Comparison of baseline characteristics of participants included in and excluded from the present study 9](#_Toc119915089)

[Supplementary table 4. Baseline characteristics of blood biomarkers and physical measurements according to four separate groups (N=12,377) 11](#_Toc119915090)

[Supplementary table 5. Summary of CA and control-derived KDM-BA-related measurements for participants according to four separate groups 13](#_Toc119915091)

[Supplementary table 6. Associations of per 1-year increment of KDM biological age acceleration with all-cause mortality stratified by sociodemographic and lifestyle factors at baseline 14](#_Toc119915092)

[Supplementary table 7. Associations of per 1-SD increment of KDM age acceleration with all-cause mortality in all participants and four separate groups 16](#_Toc119915093)

[Supplementary table 8. Associations of per 1-year increment of KDM age acceleration with all-cause mortality in all participants and four separate groups after excluding those who reported taking antihypertensive medication 17](#_Toc119915094)

**Members of the China Kadoorie Biobank collaborative group**

**International Steering Committee:** Junshi Chen, Zhengming Chen (PI), Robert Clarke, Rory Collins, Yu Guo, Liming Li (PI), Jun Lv, Richard Peto, Robin Walters.

**International Co-ordinating Centre, Oxford:** Daniel Avery, Derrick Bennett, Ruth Boxall, Sue Burgess, Ka Hung Chan, Yumei Chang, Yiping Chen, Zhengming Chen, Johnathan Clarke; Robert Clarke, Huaidong Du, Ahmed Edris Mohamed, Zammy Fairhurst-Hunter, Hannah Fry, Simon Gilbert, Alex Hacker, Mike Hill, Michael Holmes, Pek Kei Im, Andri Iona, Maria Kakkoura, Christiana Kartsonaki, Rene Kerosi, Kuang Lin, Mohsen Mazidi, Iona Millwood, Sam Morris, Qunhua Nie, Alfred Pozarickij, Paul Ryder, Saredo Said, Sam Sansome, Dan Schmidt, Paul Sherliker, Rajani Sohoni, Becky Stevens, Iain Turnbull, Robin Walters, Lin Wang, Neil Wright, Ling Yang, Xiaoming Yang, Pang Yao.

**National Co-ordinating Centre, Beijing:** Yu Guo, Xiao Han, Can Hou, Jun Lv, Pei Pei, Chao Liu, Canqing Yu, Qingmei Xia. **10 Regional Co-ordinating Centres: Qingdao CDC:** Zengchang Pang, Ruqin Gao, Shanpeng Li, Haiping Duan, Shaojie Wang, Yongmei Liu, Ranran Du, Yajing Zang, Liang Cheng, Xiaocao Tian, Hua Zhang, Yaoming Zhai, Feng Ning, Xiaohui Sun, Feifei Li. **Licang CDC:** Silu Lv, Junzheng Wang, Wei Hou. **Heilongjiang Provincial CDC:** Wei Sun, Shichun Yan, Xiaoming Cui. **Nangang CDC:** Chi Wang, Zhenyuan Wu,Yanjie Li, Quan Kang. **Hainan Provincial CDC:** Huiming Luo, Tingting Ou. **Meilan CDC:** Xiangyang Zheng, Zhendong Guo, Shukuan Wu, Yilei Li, Huimei Li. **Jiangsu Provincial CDC:** Ming Wu, Yonglin Zhou, Jinyi Zhou, Ran Tao, Jie Yang, Jian Su. **Suzhou CDC:** Fang Liu, Jun Zhang, Yihe Hu, Yan Lu, Liangcai Ma, Aiyu Tang, Shuo Zhang, Jianrong Jin, Jingchao Liu. **Guangxi Provincial CDC:** Mei Lin, Zhenzhen Lu. **Liuzhou CDC:** Lifang Zhou, Changping Xie, Jian Lan,Tingping Zhu,Yun Liu, Liuping Wei, Liyuan Zhou, Ningyu Chen, Yulu Qin, Sisi Wang. **Sichuan Provincial CDC:** Xianping Wu, Ningmei Zhang, Xiaofang Chen, Xiaoyu Chang. **Pengzhou CDC:** Mingqiang Yuan, Xia Wu, Xiaofang Chen, Wei Jiang, Jiaqiu Liu, Qiang Sun. **Gansu Provincial CDC:** Faqing Chen, Xiaolan Ren, Caixia Dong. **Maiji CDC:** Hui Zhang, Enke Mao, Xiaoping Wang, Tao Wang, Xi zhang. **Henan Provincial CDC:** Kai Kang, Shixian Feng, Huizi Tian, Lei Fan. **Huixian CDC:** XiaoLin Li, Huarong Sun, Pan He, Xukui Zhang. **Zhejiang Provincial CDC:** Min Yu, Ruying Hu, Hao Wang. **Tongxiang CDC**: Xiaoyi Zhang, Yuan Cao, Kaixu Xie, Lingli Chen, Dun Shen. **Hunan Provincial CDC:** Xiaojun Li, Donghui Jin, Li Yin, Huilin Liu, Zhongxi Fu. **Liuyang CDC:** Xin Xu, Hao Zhang, Jianwei Chen,Yuan Peng, Libo Zhang, Chan Qu.

Supplementary methods

Calculation of biological age measurement under Klemera and Doubal’s method (KDM-BA) and biological age acceleration under Klemera and Doubal’s method (KDM-AA)

$KDM-BA=\frac{\sum_{j=1}^{m} \left( x_{j}-q_{j} \right)\left( \frac{k_{j}}{s_{j}^{2}} \right)+\frac{\mathrm{CA}}{s_{\mathrm{BA}}^{2}}}{\sum_{j=1}^{m} \left( \frac{k_{j}}{s_{j}^{2}} \right)+\frac{1}{s_{\mathrm{BA}}^{2}}}$ （1.1）

m: the number of principal components (PCs)；

x_j_: the jth PC；

q_j_: the intercept of the regression of jth PC on biological age (BA), since BA is unknown, it is replaced by chronological age (CA), the same as below；

k_j_: the slope of the regression of jth PC on BA；

s_j_: the root mean square error of the regression of jth PC on BA；

s_BA_: the root mean square error of the regression of CA on BA, and it can be replaced by the root mean square error of the regression of all PCs on CA account of unknown BA,。

KDM-BA=β_0_+β_1_*CA+u （1.2）

The regression residual u is the KDM-AA we need.


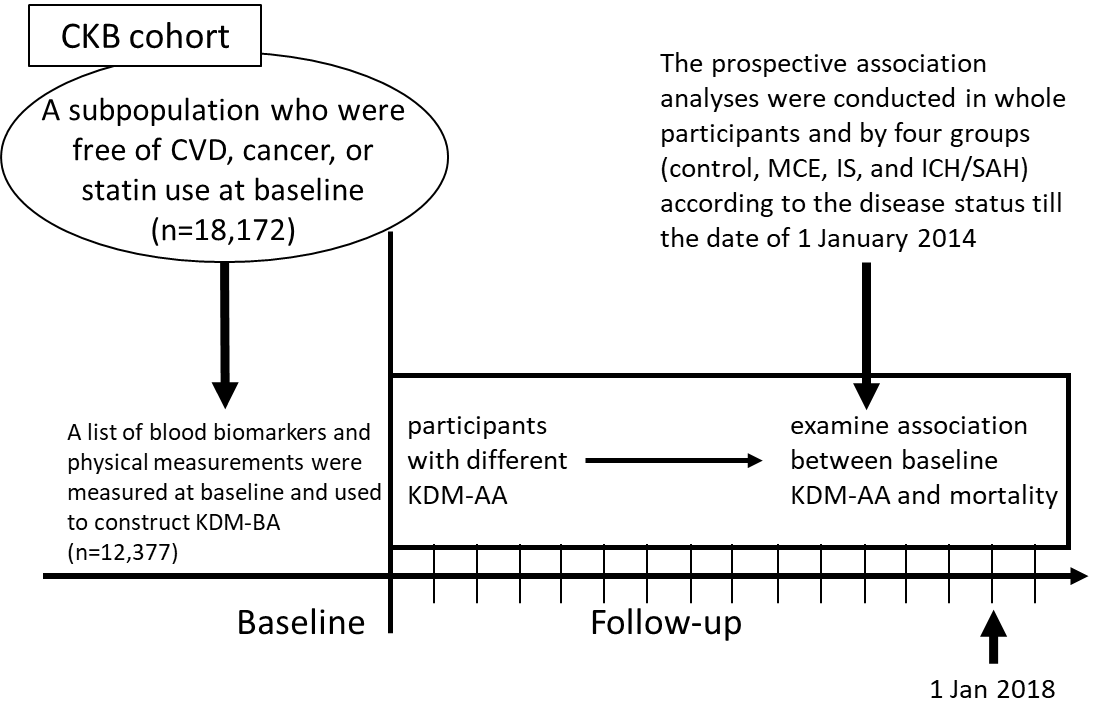


Supplementary figure 1. Study overview

Abbreviations: CKB: China Kadoorie Biobank; CVD: cardiovascular disease; MCE: major coronary event; IS: ischemic stroke; ICH: intracranial hemorrhage; SAH: subarachnoid hemorrhage; KDM: Klemera and Doubal’s method; BA: biological age; AA: age acceleration.

Supplementary table 1. Summary of reagents, calibrators, and settings in clinical biochemistry tests

| Biomarkers | Reagent | Reaction type | Analytical wavelength | Calibrator |
| --- | --- | --- | --- | --- |
| Albumin, g/L | Beckman reagent: OSR6102 | End Point | Primary: 600nm Secondary: 800nm | Beckman Coulter System Calibrator: REF 66300 |
| Alanine aminotransferase, U/L | Beckman reagent: REF OSR6107 | Rate | 340nm | Beckman Coulter System Calibrator: REF 66300 |
| Aspartate aminotransferase, U/L | Beckman reagent: REF OSR6109 | Rate | 340nm | Beckman Coulter System Calibrator: REF 66300 |
| Glutamyl transpeptidase, U/L | Beckman reagent: REF OSR6120 | Rate | 410/480nm | Beckman Coulter System Calibrator: REF 66300 |
| Creatinine, umol/L | Beckman reagent: REF OSR6*78 ^a^ | Kinetic (Jaffé Method A) | Primary: 520nm Secondary: 800nm | Beckman Coulter System Calibrator: REF 66300 |
| Cystatin C, mg/L | Siemens reagent: Kit part number OQNM175 | Immunonephelometry | N/A | Siemens N Protein Standard UY (part number OQLV05) |
| Uric acid, umol/L | Beckman reagent: REF OSR6198 | End point | Primary: 660nm Secondary: 800nm | Beckman Coulter System Calibrator: REF 66300 |
| Total triglyceride, mmol/L | Beckman reagent: REF OSR6*118 ^a^ | End point (coupled enzymatic assays) | Primary: 660nm Secondary: 800nm | Beckman Coulter System Calibrator: REF 66300 |
| Apolipoprotein A1, mg/dL | Beckman reagent: REF OSR6142 | Turbidimetric | Primary: 540nm Secondary: 800nm | Beckman Coulter Apo A1 & B Calibrator: REF ODR3022 |
| Apolipoprotein B, mg/dL | Beckman reagent: REF OSR6143 | Turbidimetric | Primary: 340nm Secondary: 800nm | Beckman Coulter Apo A1 & B Calibrator: REF ODR3022 |
| Lipoprotein A, nmol/L | Beckman reagent | Immunoturbidimetric | 700nm | Beckman Calibrators |
| Total cholesterol, mmol/L | Beckman reagent: REF OSR6116 | End point (coupled enzymatic assays) | Primary: 540nm Secondary: 600nm | Beckman Coulter System Calibrator: REF 66300 |
| High density lipoprotein cholesterol, mmol/L | Beckman reagent: REF OSR6*95 ^a^ | End point | Primary: 600nm Secondary: 700nm | Beckman Coulter HDL Calibrator: REF ODC0023 |
| Low density lipoprotein cholesterol, mmol/L | Beckman reagent: REF OSR6*96 ^a^ | End point | Primary: 540nm Secondary: 660nm | Beckman Coulter HDL Calibrator: REF ODC0024 |
| High sensitivity C-reactive protein, mg/L | Beckman reagent: REF OSR6299 | Fixed | Primary: 570nm | Beckman Coulter CRP Latex Calibrator Highly Sensitive Set: REF ODC0027 |

^a^ The * in the reagent reference code denotes a value that varies according to reagent pack size.

Supplementary table 2. Pearson’s correlation coefficients between CA and blood biomarkers or physical measurements stratified by sex

|  | Male | |  | Female | |
| --- | --- | --- | --- | --- | --- |
|  | r | *P*-value |  | r | *P*-value |
| Biomarkers |  |  |  |  |  |
| Albumin, g/L | **-0.42** | 1.20E-266 |  | **-0.20** | 3.00E-59 |
| Alanine aminotransferase, U/L | **-0.23** | 5.23E-73 |  | -0.05 | 2.55E-05 |
| Aspartate aminotransferase, U/L | 0.06 | 3.08E-06 |  | **0.12** | 6.46E-21 |
| Glutamyl transpeptidase, U/L | **-0.12** | 1.61E-22 |  | **0.12** | 5.55E-21 |
| Creatinine, umol/L | **0.18** | 1.41E-46 |  | **0.26** | 6.66E-100 |
| Cystatin C, mg/L | **0.49** | 0.00E+00 |  | **0.58** | 0.00E+00 |
| Uric acid, umol/L | 0.05 | 2.37E-05 |  | **0.25** | 6.22E-89 |
| Total triglyceride, mmol/L | **-0.13** | 3.80E-24 |  | **0.14** | 1.62E-28 |
| Apolipoprotein A1, mg/dL | **0.12** | 2.02E-20 |  | **0.19** | 1.02E-51 |
| Apolipoprotein B, mg/dL | **-0.11** | 4.05E-17 |  | **0.20** | 2.43E-55 |
| Lipoprotein A, nmol/L | 0.07 | 4.05E-08 |  | 0.06 | 6.75E-07 |
| Total cholesterol, mmol/L | -0.07 | 2.54E-07 |  | **0.21** | 2.01E-65 |
| High density lipoprotein cholesterol, mmol/L | 0.07 | 1.40E-07 |  | 0.04 | 4.09E-04 |
| Low density lipoprotein cholesterol, mmol/L | -0.05 | 7.47E-05 |  | **0.19** | 3.20E-53 |
| High sensitivity C-reactive protein, mg/L | **0.14** | 1.88E-28 |  | **0.24** | 4.13E-85 |
| Random glucose, mmol/L | **0.14** | 2.54E-27 |  | **0.16** | 1.72E-35 |
| Physical measurements |  |  |  |  |  |
| Systolic blood pressure, mmHg | **0.19** | 4.82E-51 |  | **0.24** | 8.88E-84 |
| Diastolic blood pressure, mmHg | -0.07 | 1.55E-07 |  | -0.02 | 8.50E-02 |
| Heart rate, times/min | -0.04 | 9.88E-04 |  | -0.04 | 6.47E-04 |
| Waist circumference, cm | **-0.15** | 4.21E-32 |  | 0.06 | 4.36E-07 |
| Hip circumference, cm | **-0.19** | 3.35E-50 |  | **-0.10** | 7.70E-16 |
| Waist-to-hip ratio | -0.05 | 6.68E-05 |  | **0.19** | 1.84E-53 |
| Weight, kg | **-0.32** | 4.39E-143 |  | **-0.21** | 8.23E-65 |
| Body mass index, kg/m^2^ | **-0.21** | 2.75E-60 |  | -0.08 | 1.26E-09 |
| Forced expiratory volume in one second, L | **-0.62** | 0.00E+00 |  | **-0.59** | 0.00E+00 |

Abbreviations: CA: chronological age.

All blood biomarkers were on the logarithmical scale except for albumin. For participants who reported taking antihypertensive medication (N=2,160), we added 15 and 10 mmHg to measured systolic blood pressure and diastolic blood pressure, respectively. For participants with both random and fasting blood glucose levels measured, the fasting blood glucose value was used for the analysis (N=657). Blood biomarkers or physical measurements with Pearson’s correlation coefficients ≥0.10 (displayed in bold) were used to construct biological age. Total triglyceride and hip circumference had a non-monotonic relationship with CA in women and were excluded from the construction of biological age.

Supplementary table 3. Comparison of baseline characteristics of participants included in and excluded from the present study

|  |  | Partcipants included in the present study | Participants excluded from the present study | *P*-value |
| --- | --- | --- | --- | --- |
| Number of participants |  | 12,377 | 5,795 |  |
| Sociodemographic characteristic |  |  |  |  |
| Age, year |  | 57.0 (10.5) | 57.0 (10.3) | 0.954 |
| Female, % |  | 6,218 (50.2) | 2,717 (46.9) | <0.001 |
| Urban, % |  | 3,690 (29.8) | 1,747 (30.1) | 0.647 |
| Middle school or above, % |  | 38.3 | 38.5 | 0.779 |
| Lifestyle |  |  |  |  |
| Current smoking^a^, % |  |  |  |  |
| Male |  | 69.8 | 71.3 | 0.130 |
| Female |  | 4.3 | 4.5 | 0.678 |
| Excessive alcohol drinking^b^, % |  |  |  |  |
| Male |  | 24.4 | 27.7 | <0.001 |
| Female |  | 2.0 | 2.1 | 0.840 |
| Dietary habits, % |  |  |  |  |
| Eating fresh vegetables: not daily |  | 6.8 | 6.8 | 0.961 |
| Eating fresh fruits: not daily |  | 87.2 | 87.2 | 0.944 |
| Eating red meat: daily or less than weekly |  | 48.7 | 49.3 | 0.441 |
| Eating fish: less than weekly |  | 65.3 | 65.5 | 0.722 |
| Eating soybean: <4 days/week |  | 90.8 | 90.3 | 0.271 |
| Total physical activity, MET-h/d |  | 18.6 | 17.9 | <0.001 |
| Fasting time, h |  | 4.6 | 3.7 | <0.001 |
| Prevalent hypertension, % |  | 54.5 | 57.6 | <0.001 |
| Prevalent diabetes, % |  | 6.6 | 13.2 | <0.001 |
| Medication of, % |  |  |  |  |
| Antihypertensives |  | 17.1 | 19.7 | <0.001 |
| Antidiabetics |  | 2.4 | 4.7 | <0.001 |
| Family history of, % |  |  |  |  |
| Heart disease |  | 3.4 | 3.4 | 0.833 |
| Stroke |  | 21.1 | 22.2 | 0.072 |
| Cancer |  | 15.8 | 15.2 | 0.325 |
| Self-rated good health, % |  | 40.8 | 41.2 | 0.632 |

Abbreviations: MET, metabolic equivalent of task.

Age, sex and urban regions are presented as means (SD) or n (%). Other values are means or percentages with adjustment for age, sex, and study regions, where appropriate.

^a^ Former smoker who had stopped smoking for illness was categorized into the current smoker.

^b^ Daily intake of pure alcohol: ≥30g for males, ≥15g for females; former drinker was categorized into excessive alcohol drinker.

Supplementary table 4. Baseline characteristics of blood biomarkers and physical measurements according to four separate groups (N=12,377)

|  | All | Control | MCE | IS | ICH or SAH | *P*-value |
| --- | --- | --- | --- | --- | --- | --- |
|  |  |  |  |  |  |  |
| Biomarkers |  |  |  |  |  |  |
| Random glucose (mmol/L) | 5.8 (1.6) | 5.7 (1.3) | 6.1 (1.9) | 5.9 (1.7) | 5.9 (1.6) | <0.001 |
| Albumin (g/L) | 42.1 (2.7) | 42.1 (2.7) | 42.4 (2.6) | 42.3 (2.5) | 41.7 (2.8) | <0.001 |
| Alanine aminotransferase (U/L) | 21.0 (13.4) | 20.9 (13.5) | 22.7 (13.6) | 20.6 (12.4) | 21.1 (14.2) | <0.001 |
| Aspartate aminotransferase (U/L) | 26.9 (10.6) | 27.4 (10.2) | 27.2 (11.0) | 25.1 (9.3) | 28.3 (11.9) | <0.001 |
| Glutamyl transpeptidase (U/L) | 26.1 (28.1) | 24.1 (24.5) | 29.8 (32.8) | 26.8 (27.8) | 26.8 (31.0) | <0.001 |
| Creatinine (umol/L) | 63.4 (15.3) | 63.3 (14.5) | 63.8 (15.6) | 62.0 (14.5) | 65.1 (17.0) | <0.001 |
| Cystatin C (mg/L) | 0.9 (0.2) | 0.9 (0.2) | 0.9 (0.2) | 0.8 (0.2) | 0.9 (0.2) | <0.001 |
| Uric acid (umol/L) | 271.2 (78.0) | 269.3 (76.1) | 276.2 (77.3) | 266.9 (76.9) | 277.0 (81.3) | <0.001 |
| Total triglyceride (mmol/L) | 1.5 (0.7) | 1.5 (0.7) | 1.6 (0.8) | 1.6 (0.8) | 1.5 (0.7) | <0.001 |
| Apolipoprotein A1 (mg/dL) | 134.5 (21.8) | 136.3 (21.4) | 125.2 (18.6) | 132.9 (21.4) | 136.4 (22.7) | <0.001 |
| Apolipoprotein B (mg/dL) | 83.2 (20.7) | 81.0 (19.6) | 88.2 (24.0) | 87.0 (21.2) | 80.4 (19.9) | <0.001 |
| Lipoprotein A (nmol/L) | 39.4 (48.4) | 38.3 (47.0) | 44.7 (53.5) | 39.9 (49.1) | 38.7 (47.9) | 0.004 |
| Total cholesterol (mmol/L) | 4.6 (0.9) | 4.5 (0.9) | 4.7 (1.1) | 4.7 (1.0) | 4.5 (0.9) | <0.001 |
| High density lipoprotein cholesterol (mmol/L) | 1.3 (0.3) | 1.3 (0.3) | 1.2 (0.3) | 1.2 (0.3) | 1.3 (0.3) | <0.001 |
| Low density lipoprotein cholesterol (mmol/L) | 2.4 (0.7) | 2.3 (0.7) | 2.4 (0.8) | 2.5 (0.7) | 2.3 (0.7) | <0.001 |
| High sensitivity C-reactive protein (mg/L) | 2.5 (6.6) | 2.2 (6.3) | 2.5 (4.4) | 2.3 (5.8) | 2.9 (8.1) | <0.001 |
| Physical measurements |  |  |  |  |  |  |
| SBP (mmHg) | 144.4 (27.5) | 136.5 (23.1) | 144.1 (28.1) | 143.4 (27.3) | 155.3 (29.0) | <0.001 |
| DBP (mmHg) | 83.4 (14.5) | 78.3 (12.0) | 84.0 (14.9) | 84.6 (14.3) | 88.2 (15.5) | <0.001 |
| Heart rate (times/min) | 78.5 (11.9) | 77.9 (11.8) | 79.4 (11.8) | 78.5 (11.7) | 79.1 (12.1) | <0.001 |
| WC (cm) | 80.1 (9.8) | 78.3 (9.3) | 82.0 (9.9) | 82.0 (9.8) | 79.6 (9.8) | <0.001 |
| Hip circumference (cm) | 90.1 (7.0) | 88.8 (6.6) | 91.0 (7.1) | 92.1 (7.0) | 89.2 (6.8) | <0.001 |
| WHR | 0.9 (0.1) | 0.9 (0.1) | 0.9 (0.1) | 0.9 (0.1) | 0.9 (0.1) | <0.001 |
| Weight (kg) | 58.8 (10.9) | 57.2 (10.3) | 61.1 (11.3) | 61.5 (10.9) | 57.1 (10.7) | <0.001 |
| BMI (kg/m^2^) | 23.3 (3.4) | 22.8 (3.3) | 23.7 (3.5) | 24.2 (3.5) | 23.0 (3.5) | <0.001 |
| FEV1 (L) | 2.1 (0.7) | 2.1 (0.7) | 2.2 (0.7) | 2.3 (0.6) | 1.9 (0.7) | <0.001 |

Abbreviations: FEV1: forced expiratory volume in one second; MCE: major coronary event; IS: ischemic stroke; ICH: intracranial hemorrhage; SAH: subarachnoid hemorrhage.

All values are presented as means (SD). For participants who reported taking antihypertensive medication (N=2,160), we added 15 and 10 mmHg to measured systolic blood pressure and diastolic blood pressure, respectively. In the participants with fasting blood glucose levels measured, random blood glucose values were replaced by corresponding fasting measurements (N=657).

Supplementary table 5. Summary of CA and control-derived KDM-BA-related measurements for participants according to four separate groups

|  | All | Control | MCE | IS | ICH or SAH | *P*-value |
| --- | --- | --- | --- | --- | --- | --- |
| CA, year | 57.0 (10.5) | 58.4 (11.0) | 54.2 (8.6) | 53.7 (9.4) | 59.6 (10.4) | <0.001 |
| KDM-BA, year | 57.1 (10.5) | 58.4 (11.0) | 54.4 (8.7) | 53.8 (9.4) | 59.9 (10.4) | <0.001 |
| KDM-AA, year | 0.0 (0.6) | -0.1 (0.6) | 0.1 (0.7) | 0.0 (0.6) | 0.1 (0.7) | <0.001 |
| RMSE, year | 0.61 | 0.55 | 0.68 | 0.55 | 0.67 | - |
| Pearson’s correlation coefficient between KDM-BA and CA | 1.00 | 1.00 | 1.00 | 1.00 | 1.00 | - |

Abbreviations: KDM: Klemera and Doubal’s method; CA: chronological age; BA: biological age; AA: age acceleration; RMSE: root mean square error; MCE: major coronary event; IS: ischemic stroke; ICH: intracranial hemorrhage; SAH: subarachnoid hemorrhage.

CA, KDM-BA, and KDM-AA measurements are presented as means (SD).

Supplementary table 6. Associations of per 1-year increment of KDM biological age acceleration with all-cause mortality stratified by sociodemographic and lifestyle factors at baseline

| Subgroup | No. of deaths | Mortality (per 1000 person-years) | Adjusted HR (95%CI) | *P*-interaction |
| --- | --- | --- | --- | --- |
| Age groups |  |  |  | 0.024 |
| <50y (N=3,212) | 490 | 14.3 | 1.24 (1.16, 1.32) |  |
| 50-59y (N=3,999) | 1,029 | 25.8 | 1.24 (1.19, 1.29) |  |
| ≥60y (N=5,166) | 2,454 | 55.5 | 1.17 (1.14, 1.21) |  |
| Sex |  |  |  | <0.001 |
| Male (N=6,159) | 2,289 | 40.5 | 1.19 (1.16, 1.21) |  |
| Female (N=6,218) | 1,684 | 27.3 | 1.89 (1.69, 2.11) |  |
| Urban |  |  |  | 0.366 |
| No (N=8,687) | 3,115 | 38.5 | 1.19 (1.17, 1.22) |  |
| Yes (N=3,690) | 858 | 22.9 | 1.22 (1.16, 1.28) |  |
| Current smoking |  |  |  | <0.001 |
| No (N=7,841) | 2,245 | 29.2 | 1.24 (1.20, 1.29) |  |
| Yes (N=4,536) | 1,728 | 41.7 | 1.19 (1.15, 1.22) |  |
| Excessive alcohol drinking |  |  |  | <0.001 |
| No (N=10,805) | 3,344 | 32.1 | 1.22 (1.19, 1.25) |  |
| Yes (N=1,572) | 629 | 44.3 | 1.15 (1.10, 1.21) |  |
| Risky dietary score: 4-5 points |  |  |  | 0.775 |
| No (N=8,401) | 2,458 | 29.9 | 1.20 (1.17, 1.24) |  |
| Yes (N=3,976) | 1,515 | 41.8 | 1.19 (1.15, 1.24) |  |
| Level of physical activity ≤P_50_ |  |  |  | 0.322 |
| No (N=6,083) | 1,837 | 31.3 | 1.18 (1.14, 1.22) |  |
| Yes (N=6,294) | 2,136 | 35.8 | 1.21 (1.17, 1.25) |  |
| Risky lifestyle score: 3-4 points |  |  |  | 0.058 |
| No (N=11,160) | 3,413 | 31.6 | 1.21 (1.18, 1.24) |  |
| Yes (N=1,217) | 560 | 53.4 | 1.16 (1.09, 1.22) |  |

Abbreviations: KDM: Klemera and Doubal’s method; HR: hazard ratio.

Current smokers were defined as those who reported smoking currently or ever smokers who had stopped smoking for illness. Estimated daily consumption of pure alcohol (g) was calculated based on self-reported types of drinking and corresponding daily consumption. Drinkers with immoderate alcohol intake (≥30g for males; ≥15g for females) or former drinkers were categorized into excessive alcohol drinkers. The risky dietary habits were defined according to the following criteria: eating vegetables not daily, eating fruits not daily, eating red meat daily or not weekly, eating soybean <4 days per week, eating fish not weekly. For each food item, the participant who satisfied the above criterion scored 1 point, and otherwise 0. A composite risky dietary score was derived from the sum of the score of each food item, ranging from 0 to 5. Participants who scored 4 to 5 were categorized into the risky dietary group. Physical activity was dichotomized by the age- (<50 years, 50-59 years, and ≥60 years) and sex-specific medians of total physical activity level and the lower half constitutes the risky group. The risky lifestyle score was the number of the risky lifestyle factors, ranging from 0 (healthiest) to 4 (riskiest). Participants who scored 3 to 4 were categorized into the risky group.

Cox models adjusted for sex, fasting status, and educational attainment, stratified jointly by age groups in the 5-year interval, study areas, and four groups, where appropriate.

Supplementary table 7. Associations of per 1-SD increment of KDM age acceleration with all-cause mortality in all participants and four separate groups

|  | **Primary analysis** | |  | **Sensitivity analysis 1** | |
| --- | --- | --- | --- | --- | --- |
|  | **Adjusted HR (95%CI)** | ***P*-value** |  | **Adjusted HR (95%CI)** | ***P*-value** |
| All | 1.29 (1.25, 1.33) | <0.001 |  | 1.30 (1.26, 1.34) | <0.001 |
| Control | 1.47 (1.37, 1.59) | <0.001 |  | 1.48 (1.37, 1.60) | <0.001 |
| MCE | 1.19 (1.09, 1.29) | <0.001 |  | 1.21 (1.11, 1.32) | <0.001 |
| IS | 1.49 (1.38, 1.61) | <0.001 |  | 1.49 (1.38, 1.62) | <0.001 |
| ICH or SAH | 1.23 (1.18, 1.28) | <0.001 |  | 1.24 (1.19, 1.29) | <0.001 |

Abbreviations: SD: standard deviation; KDM: Klemera and Doubal’s method; MCE: major coronary event; IS: ischemic stroke; ICH: intracranial hemorrhage; SAH: subarachnoid hemorrhage; HR: hazard ratio; BA: biological age.

Cox models adjusted for sex, fasting status, and educational attainment, stratified jointly by age groups in the 5-year interval, study areas, and four groups.

In the primary analysis, we calculated principal components (PCs) and the KDM-BA in the whole population directly. In sensitivity analysis 1, we restricted the construction of KDM-BA in the control group, and then calculated PCs and KDM-BA for the case groups using loadings of markers and weights of CA and PCs derived from the control analysis.

Supplementary table 8. Associations of per 1-year increment of KDM age acceleration with all-cause mortality in all participants and four separate groups after excluding those who reported taking antihypertensive medication

|  | **No. of deaths** | **Mortality (per 1000 person-years)** | **Adjusted HR (95%CI)** | ***P*-value** |
| --- | --- | --- | --- | --- |
| All | 3047 | 30.75 | 1.25 (1.21, 1.28) | <0.001 |
| Control | 511 | 12.29 | 1.40 (1.30, 1.51) | <0.001 |
| MCE | 471 | 96.27 | 1.15 (1.06, 1.25) | 0.001 |
| IS | 465 | 14.07 | 1.38 (1.27, 1.49) | <0.001 |
| ICH or SAH | 1600 | 81.79 | 1.20 (1.15, 1.25) | <0.001 |

Abbreviations: KDM: Klemera and Doubal’s method; MCE: major coronary event; IS: ischemic stroke; ICH: intracranial hemorrhage; SAH: subarachnoid hemorrhage; HR: hazard ratio.

Cox models adjusted for sex, fasting status, and educational attainment, stratified jointly by age groups in the 5-year interval, study areas, and four groups.
